# The paradox of radiation and T cells in tumors

**DOI:** 10.1016/j.neo.2022.100808

**Published:** 2022-06-09

**Authors:** Michael J. Gough, Marka R. Crittenden

**Affiliations:** a Earle A. Chiles Research Institute, Robert W. Franz Cancer Center, Providence Portland Medical Center, 4805 NE Glisan St., Portland, OR 97213, USA; b The Oregon Clinic, Portland, OR, 97213, USA

## Abstract

In this review we consider what appears to be a paradox in immunotherapies based around radiation therapy. The paradox is based on three main points. 1. That T cells are needed for radiation's efficacy; 2. That tumor-specific T cells are enriched in the field of treatment; and 3. That radiation kills T cells in the treatment field. We discuss evidence of the effect of radiation on T cells in the field given their ongoing movement in and out of tissues and the tumor, and how the movement of T cells impacts the treated primary tumor and untreated distant metastases. Given this evidence, we revisit the paradox to understand how the extraordinary efficacy of radiation and immunity in preclinical models is dependent on this radiation sensitive cell.

## Introduction

Radiation therapy has a conflicting interaction with T cells. Radiation has long been known to kill T cells in the treatment field. [1] This is readily observable in patients, since for approximately 40 years it has been known that traditionally fractionated radiation treatment of cancer can cause systemic lymphocyte loss. Systemic lymphocyte loss has been observed in a range of tumor types and primary tumor locations, [2], [3], [4], [5], [6], [7] and this observation has persisted to the present day despite advances in delivery methods and chemotherapy partners. [8,9] The loss of peripheral T cells can in part be alleviated by altering radiation dose fractionation. [10,11] The mechanisms that result in long term lymphopenia are unclear. In our studies, we found that lymphopenia following fractionated radiation therapy occurred despite normal levels of T cell homeostatic cytokines in the serum, and normal responses of T cells in these patients to cytokines *ex vivo*. [10] It is also unclear whether systemic lymphopenia is reflected in the tumor. Untreated patients with peripheral lymphocyte or myeloid expansions or contractions do not have correlated expansion or contractions in those infiltrates in the primary tumor. [12] In preclinical models, total body radiation has a much more dramatic effect on circulating lymphocytes than those in the tumor, [13] suggesting that systemic effects may be distinct from effects on the tumor environment. However, systemic lymphopenia is associated with worse prognosis in patients. [14] Systemic lymphopenia, which is defined as lymphocyte counts below the normal range following clinical complete blood counts, may also impact immunotherapy responses, since lymphopenia is a predictor of poor outcome with checkpoint immunotherapy. [15,16] Thus, as we will discuss further, radiation therapy can negatively impact lymphocytes in the field and result in systemic effects that can impact patient anti-tumor immunity.

Despite this negative effect of radiation on lymphocytes, over the past decade it has become accepted that a significant portion of the efficacy of radiation therapy is dependent on T cells killing residual cancer cells. Many investigators have demonstrated that immunotherapies that target T cells dramatically increase tumor control following radiation therapy in preclinical models. Thus, as recently reviewed, [17] there is extensive evidence for the importance of immune cells for tumor control in preclinical tumor models. However, that data is less clear in patients. Mechanistic studies proving the role of immune cells are not possible in people, and instead we often rely on correlative data based around circulating neutrophil: lymphocyte ratios, absolute circulating lymphocytes counts, and serum cytokines. [17] Nevertheless, critical data in patients demonstrates that immunosuppressed patients have inferior outcomes following surgery and post-operative radiation therapy. [18] In addition, there are important data from patients with accessible tumors that are commonly treated with neoadjuvant radiation that can then be evaluated for their pre-treatment environment and post-treatment responses. In rectal cancer patients, a high density of CD8 and CD4 T cells and a low density of myeloid cells prior to treatment was associated with an improved pathological response evaluated at the subsequent resection. [19] In head and neck cancer patients, a higher tumor infiltration of myeloid cells into the pre-treatment tumor was associated with poor prognosis following definitive chemoradiation therapy. [20] Similarly, in rectal cancer patients high Treg density post treatment at resection was associated with a decreased pathologic response. [21] These data are very consistent with preclinical observations where high T cell infiltrates prior to treatment are associated with good outcomes, and myeloid populations can limit outcomes following radiation therapy (reviewed in [22,23]). However, there are conflicting data in the effect of radiation therapy on the tumor immune environment between preclinical models and human patients. For example, interferon responses induced by nucleic acid sensing or T cell activity is generally a positive feature of immunotherapy and radiation therapy combinations. [24,25] Interferons can upregulate antigen presentation and processing pathways in local and distant cancer cells, [26] and have locoregional effects including direct T cell differentiation towards effector phenotypes with improved anti-tumor efficacy, [27] myeloid differentiation towards tumor-suppressing phenotypes, [28] and induce chemokines that recruit effector T cells capable of controlling tumors. [29] However, in preclinical models an IFN-related signaling was associated with resistance to RT. [30] Overexpression of Stat1 resulted in radioprotection of cancer cells, though these studies were performed in immunodeficient mice, [30] so the impact of T cell derived interferon gamma could not be addressed. T cell production of interferon gamma is critical to the immune contribution to tumor control in preclinical models. [31] A related interferon-related DNA damage resistance signature was identified in patients, and predicted a poor response to therapy in patients with breast cancer[32] and glioblastoma. [33] These issues are difficult to reconcile, since interferon gamma can have multiple positive effects on the tumor immune environment beyond the site of secretion. [26] One mechanism that may explain these data is that chronic activation of interferon signals can suppress key pathways in cancer cells. In preclinical models, chronic in vitro exposure to interferon gamma generated a cell line that had become resistant to radiation therapy and immunotherapy combinations. [34] This was associated with stable epigenetic changes in the cancer cell and can impact T cell exhaustion in the treated tumor environment. [34] This highlights a series of paradoxes in the role of IFN in the tumor environment, since for example while PD-L1 is an interferon-inducible gene in cancer cells and can indicate pre-existing immune activation in the tumor environment, [35,36] it also serves to provide negative feedback to activated T cells. [36], [37], [38] In patients with acquired resistance of PD1 blockade, the cancer cells were found to have acquired loss of function in Jak genes that are essential for interferon gamma signaling. [39] In murine models of lung cancer, cell lines that were selected for resistance to PD1 therapy through repeated in vivo passage were found to have decreased features of the antigen presentation pathway, and were poorly infiltrated by T cells [40]; however, radiation therapy was able to restore antigen presentation and improve tumor control by providing type I IFN in the tumor environment. [40] Thus, loss of interferon gamma signaling can be overcome with other innate pathways in some circumstances. High PD-L1 expression pre-treatment was linked to improved outcome following RT in head and neck squamous cell carcinoma, [41] in postoperative adjuvant RT in esophageal squamous cell carcinoma, [42] in EBV-positive nasopharyngeal carcinoma, [43] and the combination of low PD-L1 expression and high CD8^+^ TIL density was associated with a favorable survival rate in non small cell lung cancer treated with chemoradiation therapy. [44] In these patients, the PDL1 level alone was not informative for outcome, [44] which is consistent with data from The Pacific Trial where PDL1 status was not informative to predict patient outcome following treatment with PD1-PDL1 blockade and chemoradiation therapy. [45] Thus, PDL1 expression in tumors at baseline is linked to the infiltration of T cells and the effects of interferon gamma in patients, [46] and radiation therapy has been shown to increase PDL1 expression in the tumor via T cell mediated interferon gamma production, [47] and can directly increase PDL1 expression in cancer cells. [48] However, if the interferon-responsiveness of cancer cells is critical to their control by checkpoint inhibitors and radiation therapy, and T cells secreting interferon gamma are an essential element of immune control following radiation therapy, these data suggest that induction of PDL1 over the course of treatment can be a sign of success in immune modulation of the tumor, despite the data suggesting that IFN signatures are associated with poor prognosis. Together, these data suggest that the immune status in the tumor, along with the peripheral immune status, is linked to outcome following radiation therapy in patients. To directly use this information, alternative study designs may be needed. For example, Zhou *et al.* use immune parameters to identify prognostic features of outcome following checkpoint inhibitor therapy, [49] and Grass et al. have identified an immune focused gene set that associates with responsiveness to radiation therapy in patients. [50] Such radioimmunogenicity features[22] may predict outcome at baseline, but it may also be possible to dynamically follow changes in the tumor over treatment to identify responses. For example, Hecht *et al.* used induction of CD8 T cell signatures in the tumors of HNSCC patients treated with a combination of chemotherapy and checkpoint inhibitor therapy to determine whether they should go on to receive additional radiation therapy and immunotherapy combinations. [51] In this way, advanced immune monitoring can identify patients who are responding to treatment. However, this could also be used to idenitfy alternative treatment options for those who fail to respond, for example using tumor explants to identify agents that can make beneficial changes in the patient tumor, [52,53] or to identify additional therapies that may redirect outcomes in recalcitrant tumors. [54]

### Which T cells are killed by radiation therapy?

As discussed above, one of the most powerful indicators that immune mechanisms are important in patients with cancer is the correlation between immune infiltration of tumors and patient overall survival. [55,56] For example, even in pancreatic cancer, which generally has a poor infiltrate and poor outcome, the presence of T cell infiltrates into the tumor correlate with a better prognosis compared to matched patients without these infiltrates. [12,[57], [58], [59], [60], [61] In the studies cited, the patient tumors that were analyzed for their infiltrates were obtained at the time of surgical resection. Akin to the paradox of radiation and T cells, the paradox in such studies is that the infiltration of these T cells was critical to outcome, yet they were removed from the patient during surgical resection. For each scenario it is necessary to understand why the tumor environment impacts overall survival of cancer patients given their removal at resection or their irradiation.

Not all of the T cells in tumors are specific for tumor-associated antigens – in fact tumor-specific T cells may be a minor population. As we will discuss, most T cell populations recirculate throughout the body and pass out of tissues via efferent lymphatics into the peripheral blood before entering another tissue or lymphoid organ. [62] For this reason, in tumors there are broad T cell infiltrates of unknown specificity, though there are enrichments of tumor-specific T cells in the tumor. Among these tumor-specific T cells in the tumor there are also non-circulating resident T cell populations that are highly enriched for tumor specificity. [63,64] Tumor-specific T cells are very difficult to detect in the peripheral blood prior to intervention, [65] since they represent a very minor population of all the specificities present in the circulating T cell pool. Given that tumor-specific T cells are enriched in the tumor, and tumor resident T cell subpopulations may not leave the tumor, these tumor-specific cells may receive the full effect of repeated tumor dosing with radiation therapy. Yet as we will discuss, these pre-existing tumor resident T cells are critical for the full success of these combinations of radiation and immunotherapies. [13,^66^,67] Together, these issues present a paradox. T cells are needed for radiation's efficacy, yet radiation kills T cells and is focused on the tumor specific T cells. By understanding the impact on T cells in the field and the contribution of T cells outside the field, we can optimize approaches to synergize radiation therapy and immunotherapy for local and distant tumor control. We will discuss how radiation treatments depend on T cells that are present in the treatment field versus systemically circulating T cell populations to control residual disease in the treated tumor and distant disease.

#### Are different subsets of T cells more sensitive to radiation?

T cells are highly sensitive to radiation treatment. Recent analysis of a range of blood cell populations exposed to low doses of radiation therapy demonstrated that a significant increase in T cell death was detectable at 0.125Gy ex vivo radiation, and approximately half of T cells underwent apoptosis at 2Gy. [68] Similar work across a wide dose range has shown significant increases in lymphocyte apoptosis starting in the 0.05Gy-0.3Gy range, and without phagocytic clearance, T cells rapidly progressed into necrotic death at doses above 0.3Gy-0.1Gy. [69] This contrasted with myeloid populations, which were relatively radioresistant at these doses. [68,69] Patients treated with 2 × 2Gy per day of total body radiation demonstrated a 95% loss of T cells within 1 day of treatment. [68] In murine models, total body irradiation doses over 2Gy are sufficient to cause 10-fold or 100-fold decreases in T cells *in vitro* and *in vivo.* [70,71]

There are conflicting reports of the relative radiosensitivity of different immune cell subsets. In part, these may result from assay differences, whether purified cells or mixed PBMC populations treated *ex vivo*, or tumors treated *in vivo*. However, the dynamics of T cells in tumors makes in vivo assessments of radiosensitivity problematic. T cells are continuously entering and leaving peripheral tissues, including tumors, as they recirculate. [62] For example, in preclinical models, radiation therapy of the tumor has been shown to increase the proportions of T regulatory cells (Treg) in the tumor, [72] and multiple studies have shown data suggesting that these cells are more radioresistant or recover faster following radiation. [72], [73], [74], [75], [76] However, following radiation therapy of the tumor in mice, the proportion of Treg in the spleen were also increased, suggesting that the in-field effect was not critical to the change in Treg proportions. In the same study, the authors demonstrated that radiation of the leg of mice resulted in an overall decreased cellularity of shielded mouse spleens, but an increase in Treg as a proportion of cells in the spleen, even at low radiation doses. [72] These data suggest that radiation can systemically change the proportion of T cells in multiple sites – an effect that is often assumed to be specific to the irradiated site. This may directly relate to the recirculation of T cells throughout the animal. In a recent study, radiation was shown to result in a proportional increase in Treg 7 days following radiation therapy. [74] In an effort to define whether this increase in Treg in the tumor following radiation was a result of the relative radioresistance of Treg cells, rapid proliferation of Treg at the tumor following radiation, or rapid repopulation from a circulating Treg population, the drug FTY720 was utilized. FTY-720 treated mice are transiently depleted of T cells in the peripheral blood by blocking recirculation of T cells. Thus, if Treg are repopulating due to division at the tumor or are resistant to radiation, treatment with FTY-720 would have no impact on the increase in Treg seen in the tumor following radiation. However, in FTY-720-treated mice, radiation did not significantly increase Treg in the tumor. [74] These data suggest that repopulation from the periphery is critical to the Treg increase in the tumor following radiation therapy. In a model where mice were intentionally treated with total body irradiation, peripheral T cells were eliminated with increasing doses of radiation, but the tumor-infiltrating T cell population was not affected to the same degree. [13] Other sites such as the liver showed a greater loss of T cells than the irradiated tumor, and this appeared to result from a proportional loss of circulating immune cell populations. [13] These data demonstrate that multiple circulating T cell populations are negatively impacted by radiation therapy, but surprisingly those within the tumor are relatively resilient or recover quickly. To understand these data, it is important to explore where critical immune cells are at the time of treatment, and where they are most affected by radiation therapy. For the purposes of this review, we will focus on CD8 T cells, which are the dominant effector population that synergizes with radiation therapy to eliminate tumors in preclinical models. This does not ignore critical contributions of CD4 T cells. Data from infectious disease models has shown that while primary responses to infectious agents can be unchanged in the absence of CD4 T cells, secondary responses are often decreased as a result of altered formation of CD8 T cell memory. [77,78] These data suggest that the pre-existing immune status of tumor infiltrating and circulating CD8 T cell populations may be highly dependent on functioning CD4 T cells. In addition, since CD4 T cells play a critical role in the licensing of dendritic cells for the full function of CD8 T cells, [79], [80], [81] it is likely that these cells are critical components of the lymph node to support CD8 expansion. Moreover, non-Treg CD4 T cells are similarly sensitive to radiation toxicity to CD8 T cells, [71] while Treg may be more radioresistant. [75,76] Following fractionated chemoradiation of pancreatic cancer patients, non-Treg CD4 T cells were decreased in identical proportion to CD8 T cells in the peripheral circulation, and their rate of recovery was similar. [10] For this reason, we can anticipate that the impact of radiation on the tumor, lymph nodes, and peripheral blood will similarly impact non-Treg CD4 T cell populations and CD8 T cells, and that loss of CD4 T cells alongside CD8 T cell may directly or indirectly impact tumor control.

### Which T cells are in the treatment field?

#### Focal radiation treatment of T cells in the tumor

**Fig. 1:**
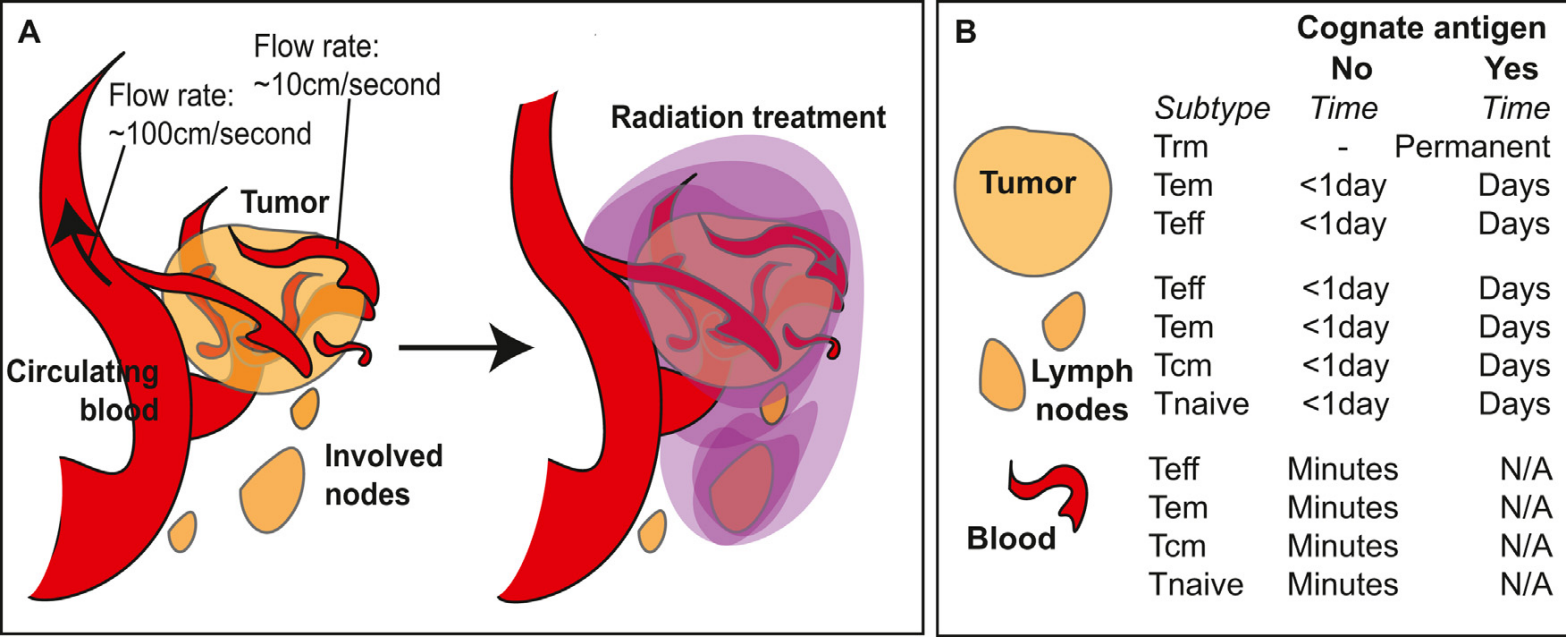
T cells within radiation treatment fields. A) With radiation treatment made up of multiple overlapping fields focused on the tumor and involved lymph nodes, cells moving through the peripheral blood inside the treatment field will also be irradiated. The flow rates of cells through these vessels means that a large proportion are likely to pass beyond the field before treatment is complete. B) Subtypes of CD8 T cells within the tumor, lymph nodes, and blood, and estimates for the time these cells spend in each site depending on whether or not they meet their cognate antigen.^62^^,^^107^

Recirculation is a critical feature of T cell biology that ensures constant scanning of peripheral sites for cognate antigen. Antigen-experienced T cells can leave the peripheral blood and enter peripheral tissues, and if they fail to meet their cognate antigen they can return to the peripheral blood via the draining lymphatics. [62] This is an ongoing process that takes T cells through multiple tissues and lymphoid organs as part of systemic immunosurveillance. Cells that meet their cognate antigen can halt recirculation and become enriched at a site (Fig. 1). For these reasons, tumors are enriched for tumor specific T cells. In addition, critical subpopulations of tissue resident memory cells (Trm) can also be enriched in the tumor. [63,64] These are more likely to be tumor-specific, and patients with more of these cells have improved outcomes. [63,64] In non-cancer settings, these Trm can provide rapid local response to repeat infection, and hence represent a critical adaptive immune defense to recurrent pathogens. Their presence in the barrier site provides local specific immunity, and their phenotype includes features that allow tissue residency and prevent recirculation. [82], [83], [84] Similar phenotypic features have been identified in tumor Trm, and these cells have minimal shared TCR homology with circulating T cells. [63,85] In murine tumor models we can observe T cells specific for tumor associated antigens in the tumor including tumor-specific T cells with Trm phenotypes, but as the tumor progresses the tumor-specific T cells become difficult to detect in the peripheral circulation. [66]

Yet, despite the unique properties of T resident memory cells, it is generally assumed that there are other CD8 T cells with tumor specificity that are circulating in the patient. [62] The issue is that these cells are infrequent enough to be below the detection threshold of conventional T cell assays. [65] In preclinical models of immunity to viruses, T cells specific for the same antigen can be found as both Trm and as classical circulating memory populations. [86] However, clonotypic analyses of tumors suggests that the Trm TCR clonotypes are relatively unique to tumors and poorly shared with T cells in the draining lymphatics and the peripheral blood. [63] In normal tissues, Trm induced by initial antigen exposure can be induced to recirculate as classical memory cells by repeat antigen exposure. [86] In tumors antigen is chronically present, which may differently impact Trm biology. For example, Trm in tumors exhibit exhaustion markers that are not seen in normal tissue Trm. [63,^64^,87] Given the exhausted state of these T cells, it is unclear whether chronically antigen exposed tumor Trm can be driven to recirculate. [62] In infectious disease models, rechallenge experiments have also shown that pre-existing Trm can become dominant at an infection site, such that new antigens present in infections do not effectively displace existing Trm with Trm with new specificities so long as the earlier antigen is also present. [88] These data suggest that in the tumor where antigen is chronic, existing Trm may hold their unique position so long as their antigen remains.

Recent data has demonstrated that Trm are equally sensitivity as other T cell populations in the tumor following radiation treatment. [13,66] In each study, Trm were reduced in number following radiation therapy, but their proportion to other T cell populations in the tumor were unchanged, [13,66] suggesting that these cells are not differentially sensitive to radiation therapy. While tumor radiation therapy decreases the number of T cells in the tumor, the proportion of these that are Trm is not changed. [13,66]

Recently, we demonstrated that Trm are critical to tumor control by radiation therapy and immunotherapy combinations. [66] If these cells are enriched in the field and their presence is critical yet they are killed by radiation therapy, what role can these cells play? Firstly, they are not all killed by radiation. [13,66] Secondly, in their role as sensors of local antigen, very small numbers may be sufficient. In normal tissues Trm play a critical response in the rapid response to reinfection. These cells are sensitive to their target antigen and can direct recruitment of additional immune populations to control the infection. [82], [83], [84] It is possible that they play a similar role in the irradiated tumor. However, the impact of T cells can vary quite dramatically between tumor models. For example, the MC38 colorectal carcinoma and Panc02-SIY pancreatic adenocarcinoma models were shown to be equally radiosensitive in vitro; however, in vivo the MC38-tumor was highly responsive to 12Gy radiation therapy, while the Panc02-SIY tumor exhibited a modest growth delay when treated at this dose. [89] Following depletion of CD8 T cells, much of the benefit of radiation treatment of the MC38 tumor was removed, and these tumors now exhibited only a modest growth delay following radiation therapy, while the modest Panc02-SIY response to radiation following CD8 T cell depletion was unchanged. [89] Matching results were obtained with the more related Moc1 and Moc2 oral carcinoma models, [90] where the two cell lines exhibited identical in vitro radiosensitivity and very different in vivo responses to 12Gy radiation therapy, with the difference entirely due to the action of CD8 T cells. [90] These data, like many similar experiments in the literature demonstrate that where T cells contribute to tumor control following radiation therapy that cancer cell death is likely the initiating event, but it need not be the major impact on the tumor environment to result in long term cure. Tumor downregulation of antigen presentation via MHCI results in checkpoint blockade resistance in human patients. [39,91] The expression level of MHC-peptide on target cells impacts the effectiveness of CD8+ T cell responses, [92] where increasing density of MHC and peptide results in increased proliferation and increased cytotoxic function. [92], [93], [94] Following radiation therapy, cancer cells can upregulate MHCI and other features of antigen presentation via a range of mechanisms. [95], [96], [97], [98], [99] While the tumor is growing the Trm may be in an equilibrium state but following upregulation of MHCI or downregulation of negative signals, it is possible that the remaining Trm can be reactivated and generate IFNg. In infectious sites, this is the critical signal to drive recruitment [82], [83], [84]. Similarly in tumors, IFNg expression by T cells is critical for tumor control following radiation therapy. [31] In this way, the smaller population of Trm that survive initial radiation treatment may play a critical role in recruiting new T cells into the treatment site, or providing cytokine signals such as IFNg that dictate tumor control rather than permit the development of suppressive repair responses in the tumor following treatment. [100] This recruiting role may not only apply to Trm. While Trm may exist in close association with cancer cells, it is reasonable to think that all tumor-specific T cells that are present in the tumor following radiation therapy and manage to survive the treatment can help recruit new T cells through secretion of inflammatory cytokines and chemokines.

#### Incidental radiation treatment of T cells passing through the field

In addition to the tissues in the field, radiation will also be delivered to cells in the blood pool passing through the beam. Given the high rate of blood flow through large vessels, their presence in the field has the potential to impact large numbers of blood cells. However, this flow rate means that each cell spends a very small period in the field (Fig. 1). Flow rates are highest in larger arteries, with the thoracic aorta having velocities above 150cm/second and the abdominal aorta above 100cm/second. [101] For smaller vessels, arterial flow rates were calculated at 4.9-19 cm/second. [102] For larger veins, flow rates of above 10cm/second are measured in the vena cava and other large veins[101] and in smaller veins flow rates were measured at 1.5-7.1 cm/second. [102] Since modern IMRT consists of a series of shaped fields from a range of treatment angles lasting up to a few seconds each, these flow rates mean that few cells in the peripheral arterial and venous blood will receive a full dose during a single treatment as they are travelling too fast to stay in field. Nevertheless, based on a 10cm/second flow rate through a brain treatment field, it was calculated that after a typical GBM treatment course consisting of 60 fractions of 2Gy to an 8cm field, 98.8% of all blood receives at least 0.5Gy radiation. [7] However, these data assume a constant presence for T cells in the peripheral blood. In fact, T cells spend very little time in the blood. Estimates suggest only approximately 2% of T cells are found in the peripheral blood, [103] much larger numbers are found in peripheral tissues and 10-20 fold higher numbers are found in the peripheral lymphoid organs than the blood. [103,104] This is confirmed by recent studies analyzing specific T cells in models of long-term memory to viral infection, where less than 4% of the virus-specific T cell memory population was present in the peripheral blood at any one time. [82] Classic studies based on the output from the thoracic duct demonstrated that the total blood pool of lymphocytes can be refreshed 11 times per day. [105] Modeling studies demonstrate that T cells may spend only 1-2 circulations through the heart, which corresponds to timescales of minutes in the bloodstream. [106,107] This compares to approximately 10 hours T cells spent in a lymph node. [107,108] The long periods spent outside the circulation and the random placement of small proportions of T cells in the blood pool for any one treatment, over a multi-week course of daily radiation therapy it becomes much less likely that these cells will receive significant doses based on overall blood pool exposure. Despite these questions, field size is a strong correlate of lymphocyte loss over the course of fractionated radiation therapy. [9,109] The dose to secondary lymphoid organs such as the spleen can impact lymphocyte loss, [109] though loss still occurs when the treatment is delivered to a location where the spleen is not involved. It remains to be determined whether the inclusion of lymph nodes in the treatment plan is associated with loss of lymphocytes, [110] especially for those locations where spleen dose is not applicable. Interestingly, the loss in lymphocytes after the first fraction is a potential predictor of overall lymphocyte lossm, [109] indicating that patient or plan-specific features dictate the impact to circulating immune cells that will be revealed over the course of treatment. Lymphocyte sparing treatment plans that treat large blood vessels as organs at risk may play an important role in evaluating blood pool radiation as a mechanism of treatment-related lymphopenia in patients. [9]

**Fig. 2:**
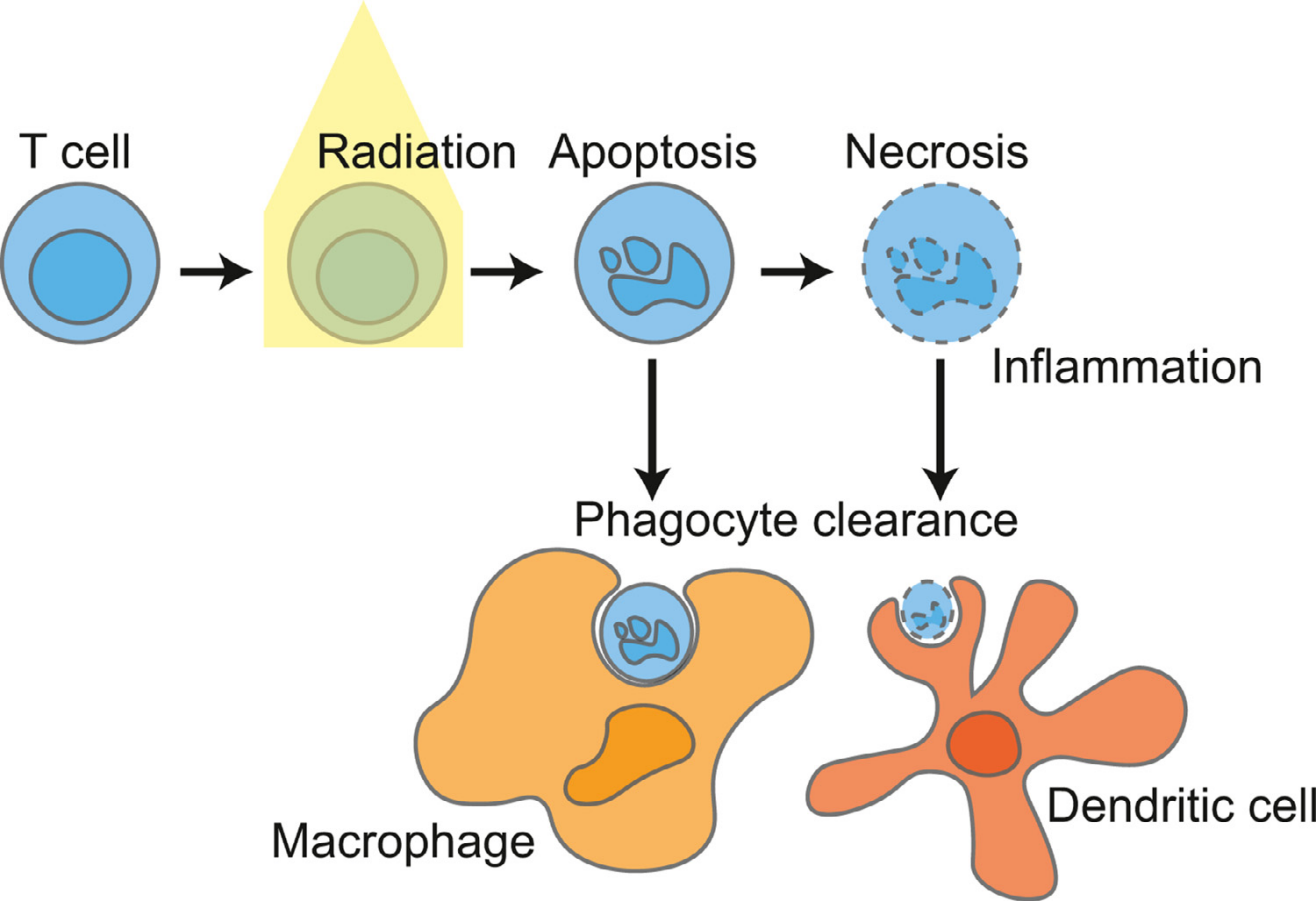
Clearance of irradiated lymphocytes. Radiation treatment of T cells results in cell death, which in turn results in phagocytic clearance of the dying cells. Circulating cells are actively cleared by macrophages in the marginal zone of spleens, though a range of phagocytic cells have the capacity to phagocytose these cells, and local phagocytic mechanisms likely drive clearance of irradiated T cells in tissues and tumors. Failure of macrophage clearance can result in inflammatory modes of death, and uptake from less abundant phagocytic cells such as dendritic cells which may impact the immune response to cell death. Systemic administration of apoptotic lymphocytes can result in systemic immune suppression, though it is unclear whether this occurs following lymphodepleting radiation treatments.

The routes for uptake of dying lymphocytes have been well described. For circulating lymphocytes, the marginal zone of the spleen is an important site of uptake of apoptotic lymphocytes. [111,112] The location of the phagocytic cells can be critical to their role in clearance. For example, in areas where large numbers of lymphocytes are known to die, such as the thymus or germinal centers of lymphoid organs, critical macrophage populations are present to remove these cells. [113] In the spleen, the marginal zone macrophages can limit availability of these cells to other phagocytic population that could otherwise take up these cells. [114,115] Within tissues, macrophages are also potent phagocytic cells, and this may be even more important in the tumor, where macrophages may be more abundant and dying cells more frequent. [100] Macrophages have been shown to be an important cell in the uptake of apoptotic lymphocytes, [114,115] for example this represents an important mechanism by which HIV-infected CD4 T cells can transmit the virus to macrophages in vitro. [116] The mechanisms of lymphocyte uptake are very similar to those involved in uptake of irradiated cancer cells [100,^117^,118] (Fig. 2). Importantly, apoptotic cell uptake by macrophages results in immune suppression, [119,120] and is mediated by specific phagocytic receptors on macrophages such as Mertk. [121] Mertk-mediated uptake of dying cells can impact immune suppression systemically [122] or in the irradiated tumor. [123,124] It is unclear whether the large-scale clearance of dying lymphocytes contributes to the negative impact of systemic lymphopenia on patient outcome; however, clearance of dying cells is generally rapid and immune effects are transient, [113] so it appears more likely that long term effects relate to the lack of key lymphocytes rather than systemic suppression resulting from the transient phagocytosis.

There are techniques that can estimate the proportion of lymphocytes in a patient that have been irradiated. The presence of p-H2AX foci is a sensitive marker for DNA double strand breaks and indicates that cells have received DNA damage in a short period prior to analysis. [125] Using quantitative analysis of H2AX foci in the peripheral blood of patients within 30 minutes of radiation treatment, the data indicated that approximately 0.5% of lymphocytes show evidence of increased DNA damage over baseline (greater than 3 foci/cell). [126] Larger proportions of lymphocytes receiving significant doses have been observed in prostate cancer treatment and this can be linked to the field size and the technique used for treatment delivery. [126], [127], [128] In patients treated with radiation for non-small cell lung cancer, the proportion of lymphocytes with greater than 6 H2AX foci per cell increased from 2.5% pre-radiation, to 10% within an hour of radiation [129] indicating that large proportion of cells were in field during this treatment approach and were still in the peripheral circulation at harvest. Given that sampling happened 1 hour after treatment, [129] and that lymphocyte transit times through lymph nodes is approximately 10 hours, [107,108] these irradiated cells are unlikely to have reached the peripheral blood via recirculation from the tissues, through the lymphatics and into the blood via the thoracic duct, so must have been in the peripheral blood at the time of treatment and still present at sampling. These data suggest large numbers of peripheral blood cells are irradiated as they pass through the treatment field. These studies challenge the prior notion that T cells spend little time in the blood, though we do not know much information about the subtypes of lymphocytes that are being assessed. In these studies, the increase in H2AX foci correlated with the mean lung dose, [129] an organ that of course has a constant high-volume passage of peripheral blood cells. *Ex vivo* calibration of H2AX foci using PBMC to link the radiation dose to the number of H2AX foci per cell suggests that these lymphocytes received low doses while passing through the field [126,129]; however, these doses are within the range that can result in T cell death *ex vivo.* [68]

Together, these data suggest an unclear role for blood-pool radiation therapy in post-radiation lymphopenia. The loss of lymphocytes following treatment of larger volumes and the inclusion of secondary lymphoid organs such as the spleen in the field can result in a measurable and impactful decrease in systemic immune function following treatment. However, given the very small proportion of the peripheral blood lymphocyte population that demonstrates tumor specificity compared to the enrichment in the tumor environment, [65,130] it is likely that much of the impact of out of tumor radiation is to T cells that are specific to non-tumor targets. Despite this, patients who lose a larger proportion peripheral lymphocytes have poorer overall survival. [4,^8^,^14^,131] These data imply circulating immune cells are critical for effective immune control of tumors or are a critical readout of immune function in treated patients. In either case, irradiating a larger field appears to impair immune control of tumors in patients via its effect on lymphocyte loss.

#### Intentional or incidental treatment of T cells in lymph nodes

Tumor draining lymph nodes are commonly included in tumor treatment plans due to the high risk of cancer metastases through these sites. Where imaging or biopsy indicates cancer involvement in lymph nodes, these and potentially other lymph nodes are included in the resection or radiation plan according to the tumor type and location. Clinical studies have indicated a clear benefit to irradiating lymph nodes draining the extended tumor site to improve patient outcome, across a range of malignancies. [132,133] Yet as we will discuss, these lymph nodes are the most likely site where new immune responses can be initiated, and existing responses boosted.

**Fig. 3:**
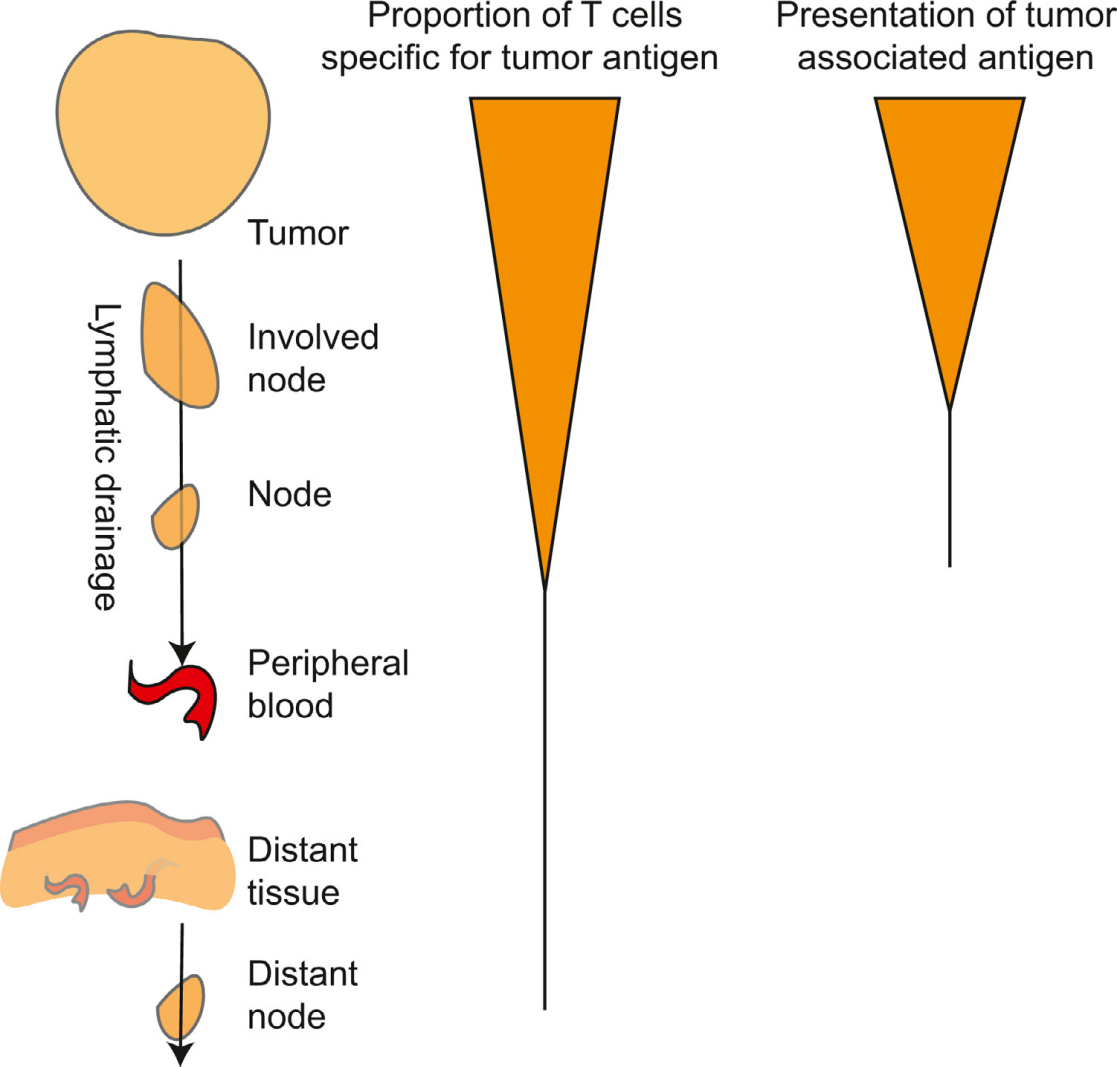
Accumulation of tumor specific T cells at sites of antigen. Antigen is abundant in the tumor and can be directly presented by cancer cells or cross presented by dendritic cells. Activated dendritic cells traffic through the efferent lymphatics to draining lymph nodes, and can cause an accumulation of specific T cells in these sites by arresting their traffic and driving expansion. Tumor specific T cells may randomly recirculate through distant sites but would be expected to rapidly exit without meeting their cognate antigen.

**Fig. 4:**
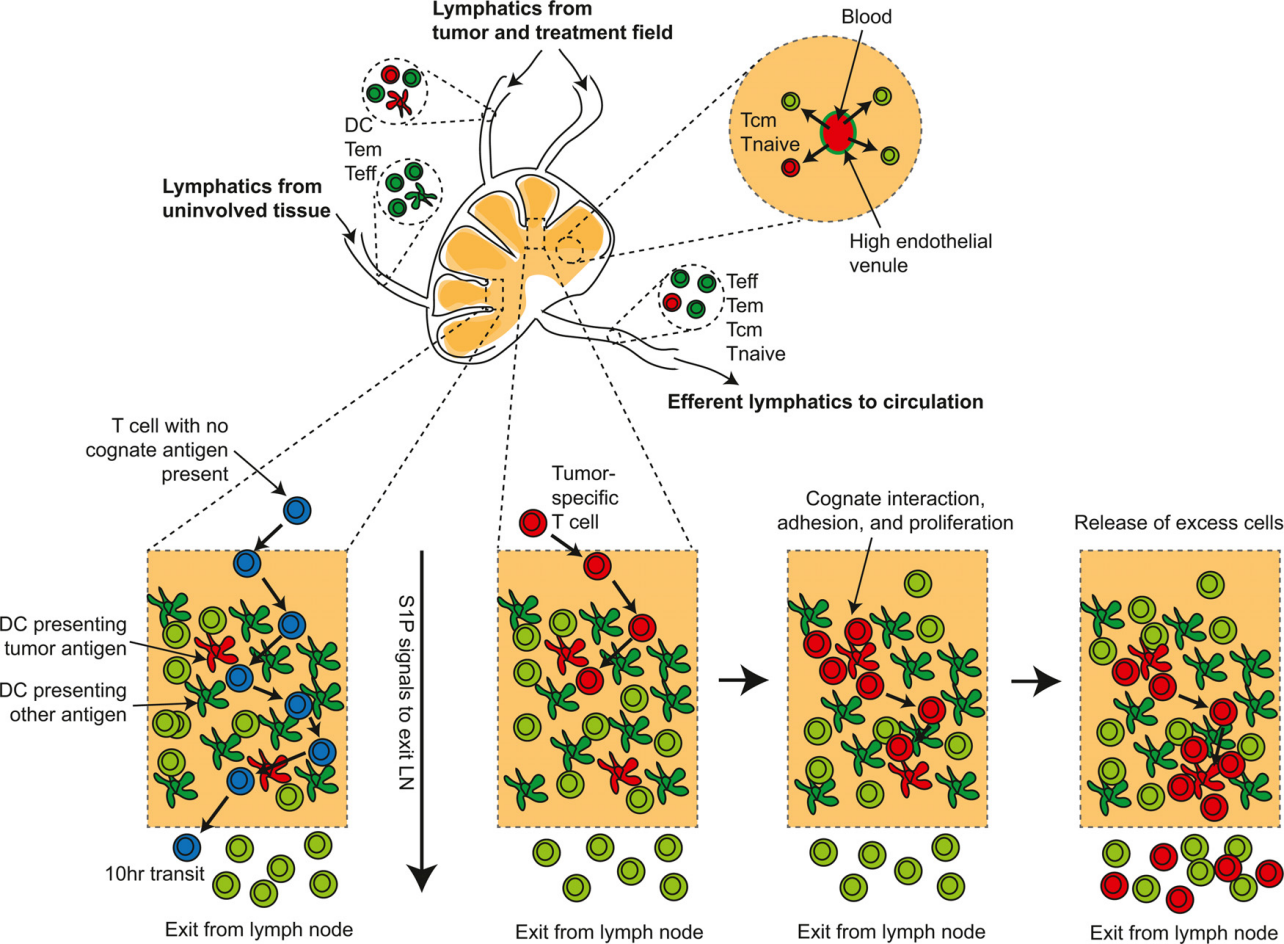
Model of tumor antigen-specific T cell accumulation in tumor-draining lymph nodes. Lymphatics draining tumors deliver T cells (effector T cells – Teff and effector memory T cells (Tem) along with dendritic cells (DC) cross-presenting tumor antigens to the draining lymph node. Other sites in the lymphatic drainage basin may also provide T cells and DC cross-presenting irrelevant antigens. In addition, naïve T cells (Tnaive) and central memory T cells (Tcm) can directly enter the lymph node via high endothelial venules. Within the lymph node T cells that fail to find their cognate antigen (blue), randomly screen antigen presenting cells under the competing influence of chemokines such as CCL7 and S1P, which directs lymphocyte exit. Tumor-specific T cells (red) may arrest on meeting their cognate antigen, and adhesion molecule interactions will overcome exit signals to permit the cells to accumulate and potentially proliferate. Over time, these cells may exceed the available antigen presenting capacity and respond to exit signals. Thus, the transit time of tumor-antigen specific cells may be significantly slower than non-specific T cells.

The unique importance of the tumor draining lymph nodes in anti-tumor immunity is due to the unique presence at this site of antigen-presenting cells cross-presenting tumor antigens (Fig. 3). These cells present antigens that originated in tissues upstream in the lymphatic drainage basin, [134,135] which may include tumor antigens. Priming of new responses by tumor-specific CD8 T cells requires dendritic cells such as conventional type 1 DCs (cDC1s) to cross-present cell-associated tumor antigens. [134,^136^,137] While other cells such as macrophages and B cells can present antigen from other cells, this antigen is presented on MHCII to CD4 T cells. [138] As with all other cells, these cells can only present their own antigens on MHCI. Dendritic cells are uniquely able to cross-present antigen from external sources to CD8 T cells. [139,140] Therefore, tumor specific CD8 T cell priming will only occur in lymph nodes containing DC that have migrated from the tumor. Trafficking of these cells from the tumor to the lymph node occurs following their maturation via CCR7-directed chemotaxis. [141] Once they have reached the lymph node, the migratory cDCs may either themselves cross-present antigens to antigen-specific CD8 T cells or pass on antigen to other cDC1 subsets in the lymph node. [141,142] We observed that in radioimmunogenic tumors that generate strong T cell control of residual disease following radiation therapy, radiation drives intratumoral cDC1 maturation. [89] However, in poorly radioimmunogenic tumors that are not supported by immune responses following radiation therapy, intratumoral cDC1 maturation did not occur following radiation therapy. [89] This migration of cDC1s from the tissue to lymphoid organs is critical since naïve T cells and central memory T cells will not be trafficking through the tissue site since only effector and effector memory T cells have the correct adhesion molecules to enter tissue sites, including the tumor (Fig. 4). The naïve and central memory T cells instead move from the peripheral blood to lymphoid organs by direct entry via CD62L interaction with high endothelial venules[83] (Fig. 4). This feature of DC biology that drives migration only to draining lymph nodes makes the tumor-draining lymph nodes critical sites of tumor antigen presentation and focal points to control ongoing immunity (Fig. 4). Since each lymph node samples and presents antigen from its upstream drainage basin, lymph nodes that drain the tumor are enriched for cross-presentation of tumor-associated antigen (Fig. 3).

Recognition of antigen in the tumor-draining lymph node can cause accumulation of antigen specific T cells (Fig. 4). Retention in a lymph node is mediated by opposing forces of chemokine-mediated attraction to other immune cells, activation-induced cell-cell adhesion, and Sphingosine-1-phosphate receptor 1 (S1PR1) -mediated attraction to the exit. [143,144] This ongoing pull via S1PR1 on the T cells and the ligand S1P that is present in the lymphatics results in egress of T cells that fail to meet their cognate ligand or have disengaged from antigen presenting cells. [143,144] Ongoing chemokine signals can overcome the drive to recirculate, where for example CCR7 on naïve T cells and memory T cells can hold T cells in lymph node locations despite S1PR1 signals. [145] T cells that meet their cognate antigen can be held in place via adhesive interactions through activation markers such as CD69, [146] which may hold cells to components of the extracellular matrix. [147] CD69 induced by exposure to type I IFN can inhibit responses to S1P, [148] and so hold activated T cells in the lymph node. CD69 is rapidly upregulated on T cell activation, but then rapidly downregulated, [146] meaning that continued activation is necessary to hold T cells in place. CD69 is a common marker of resident memory cells in tissues [149]; however, resident cells generally downregulate S1PR1, [150] resulting in a loss of exit signals that would normally drive these cells to leave their tissue or lymph node site. Thus, while resident T cells and T cells that are interacting with antigen presenting cells can be held in the lymph node, all other T cells will be encouraged to exit the lymph node and recirculate (Fig. 4).

The dynamics of this process of lymph node retention and exit can impact how radiation of lymph nodes affects immunity. If as discussed earlier, a random T cell takes approximately 10 hours to pass through a lymph node, it may only be present for one fraction of radiation. By contrast a T cell that engages with its antigen may be present for multiple fractions, and may also be proliferating, rendering these cells more susceptible to radiation-induced cell death. Moreover, any T cells that have been expanded in number as a consequence of radiation therapy may be impacted by continued treatment with fractionated radiation. [1] At present this effect is unclear, since there is contrasting data. Initial studies by Filatenkov et al. demonstrated that additional radiation of the tumor site with 3Gy x10 following an initial immunogenic 30Gy dose, resulted in impaired tumor control. [151] By contrast Savage et al. demonstrated that additional treatment with 0.5Gy x4 following an immunogenic 22Gy dose resulted in improved tumor control. [152] This was associated with modulation of suppressive immune populations that are recruited to the irradiated tumor. In addition, Zhang et al. demonstrated that two different fractionation schemes (9.18Gy x3 or 6.43Gy x10) that preserved the overall BED given to the tumor (BED_10_ of 53Gy) [153] performed equally well in combination with PD1 blockade, [154] despite the longer course of treatment extending into the effector phase of the immune response in the treatment site and potentially delivering lymphodepleting radiation doses to the key cells. These data suggest that the impact of radiation on effector-phase responses may be complex given the range of cells in the treatment site. Recently a series of studies have highlighted the potential negative impact of radiation therapy to lymph nodes on local and systemic anti-tumor immunity. In mice treated with tumor radiation therapy, the addition of tumor-draining lymph node radiation resulted in decreased tumor control. [155] Similarly, in mice treated with tumor radiation therapy combined with anti-CTLA4 treatment, the addition of tumor draining lymph node radiation resulted in decreased tumor control. [155] However, elective lymph node radiation did not impact local responses following PD1 treatment and radiation therapy. [155] The fact that lymph node radiation is less relevant where PD1 therapy is also provided is consistent with the fact that PD1 therapy can control irradiated tumors when lymph node emigration is blocked. [66] It is likely that each immunotherapy will have an optimum timing according to its mechanism of action, [156] but the variable impact of lymph node radiation on the response to PD1 and CTLA4 blockade[155] suggests understanding the dynamics of lymphocytes between sites is critical. These features should be considered when including tumor-draining lymph nodes in the field when designing radiation and immunotherapy combination clinical trials. [17]

#### T cells resident in unirradiated metastatic sites

In many cancers, overall survival following primary tumor treatment is most affected by tumor recurrence in the form of metastases. There are well-documented cases of dormant metastases re-emerging on immune suppression in patients, for example in transplant patients where tumors unknowingly present in the donor organs can emerge in the transplant recipient. [157] Preclinical data strongly suggests that both primary tumorigenesis and metastases is closely regulated by immune cells. In preclinical primary tumorigenesis models, following administration of a chemical carcinogen many mice developed rapidly progressing tumors; however, some formed small non-progressing stable masses. [158] Depletion of T cells in mice with stable masses resulted in progressively growing tumors in approximately half of the mice, suggesting that in these mice their tumor progression had been controlled by adaptive immunity. The tumors that emerged following immune suppression remained more ‘immunogenic’ than the early progressing tumors, since they were less able to form tumors on transfer to immune competent mice. [158] In preclinical genetically driven spontaneous melanoma models, metastatic spread has been shown to occur very early in tumorigenesis even though it may take hundreds of days for the tumors in some of these locations to become superficially apparent at a necropsy. [159] Depletion of CD8 T cells resulted in increased growth of visceral metastases but no increase in the number of metastases, indicating that these metastases had already seeded and were being controlled by immune cells. Together, these data suggest that some patients may have late failure of immune control that may result in emergence of primary and metastatic tumors.

Chen et al. [131] recently demonstrated that a low lymphocyte count following combination treatment with radiation therapy and a selection of immunotherapies is associated with poor impact on distant, unirradiated tumors. Interestingly, a low lymphocyte count *prior* to treatment was similarly informative on distant responses, [131] as has previously been shown for immunotherapy alone. [15] This may indicate that some level of general immunological fitness is part of the response calculation. However, although pre-treatment lymphocyte count impacted distant tumor responses, only the post-radiation lymphocyte count was associated with overall survival in patients treated with radiation therapy and immunotherapy. [131] While patients with low circulating lymphocyte counts before therapy could be considered immune deficient, the fact that the decrease in lymphocytes following radiation is most impactful to outcome suggests that recirculation of T cells to metastatic sites is important in distant tumor control following radiation therapy. These data are consistent with recent data showing that elective radiation therapy of the lymph nodes can limit distant tumor control following PD1 blockade and radiation therapy, [160] by targeting the progenitor-like memory CD8 T cells that are critical to the PD1 treatment response. [161,162] Similar studies exploring the optimum timing of radiation in combination with immunotherapy demonstrated that PD1 therapy could be given at a range of timings relative to radiation and improve local tumor control following radiation therapy. [163] However, providing PD1 therapy prior to radiation therapy mobilized a population of T cells to the tumor that were then killed by radiation therapy. [163] This correlated with a loss of distant tumor responses by the treatment, which could be avoided by providing PD1 therapy following radiation rather than prior to radiation. [163] Notably, as with prior studies, [155] local control was not affected by the alternate timing. [163] These data suggest that mobilization of systemically circulating T cell populations into the treatment field can have negative consequences for systemic immunity to tumors. Thus, the tumor draining lymph node and circulating T cell populations may contribute differently to outcome depending on whether the treatment given is radiation alone or radiation along with checkpoint inhibitors. [66,155] In addition, the impact of the lymph node and circulating T cell populations may vary depending on whether responses are measured at the site of radiation treatment or at the location of distant unirradiated disease. [160,163] The data discussed above suggests that local radiation therapy amplified by checkpoint inhibitors can control the irradiated tumor without the contribution of lymph node responses, [66,^67^,155] but to amplify systemic anti-tumor immunity following local radiation therapy, lymph node responses are important. [160] For these reasons, combination therapies that are designed to target distant disease may require different immunotherapies from those designed to control in field residual disease. For example, local administration of innate adjuvants can boost tumor-specific T cell responses in combination with radiation therapy, and these can help in control of distant disease. [164,165] However, the combination with PDL1 blockade can overcome ongoing resistance at distant sites that were not irradiated or treated with adjuvant. [165] Thus, since checkpoint inhibitors can circulate systemically and assist in T cell function in distant tumors, as has widely been proposed they may represent a final common therapy to extend local treatments to provide distant tumor control. [166,167]

If radiation therapy serves primarily as a vaccination event, then it would be possible to replace radiation therapy with tumor-specific vaccination. However, vaccination-based therapies have not significantly impacted clinical treatment of patients. [168] While vaccination effects by radiation have been observed, [169] a range of data suggest that this response alone is inadequate to generate sufficiently effective T cells for tumor control. We have found that tumor control by radiotherapy and checkpoint inhibitors depends on regulation of pre-existing immune responses rather than by vaccination by tumor irradiation. [66] In the preclinical models we commonly use to test radiation effects, the implantation of cancer cells can induce CD8+ T cell priming sufficient to establish T cell memory and can cause spontaneous rejection of immunogenic tumors. [66,^136^,[170], [171], [172] Importantly, where pre-existing anti-tumor immunity is blocked radiation therapy and checkpoint therapy fail, [66] and where implantation techniques are used to avoid pre-existing anti-tumor immunity, additional vaccination is necessary to permit tumor control by radiation and checkpoint inhibition. [173] In a side-by-side comparison of radiation therapy versus vaccination, we found that extremely potent vaccines were less able than radiation therapy to result in tumor control when combined with checkpoint blockade. [95] Relatedly, Rükert et al. demonstrated that radiation therapy combined with PD1 inhibition could impact growth of the irradiated tumor and a distant unirradiated tumor, but simultaneous vaccination did not have any further impact on tumor control. [174] Notably, most of the changes in immune infiltration occurred in the irradiated tumor, and a limited number changes occurred at the non-irradiated distant site even with the addition of vaccination. [174] In the irradiated tumor radiation has been shown to increase direct antigen presentation by the cancer cells which is critical for tumor control, [95] and also increases PD-L1 on cancer cells [175] and other immune cells in the tumor environment [174,176] which limits the activation threshold of T cells. [177] Thus, radiation therapy combined with PD1 blockade has the ability to increase ability of T cells to control the irradiated tumor, and also permits these cells to impact distant tumors, though the absence of direct radiation effects at distant sites can limit their overall capacity to cure.

**Fig. 5:**
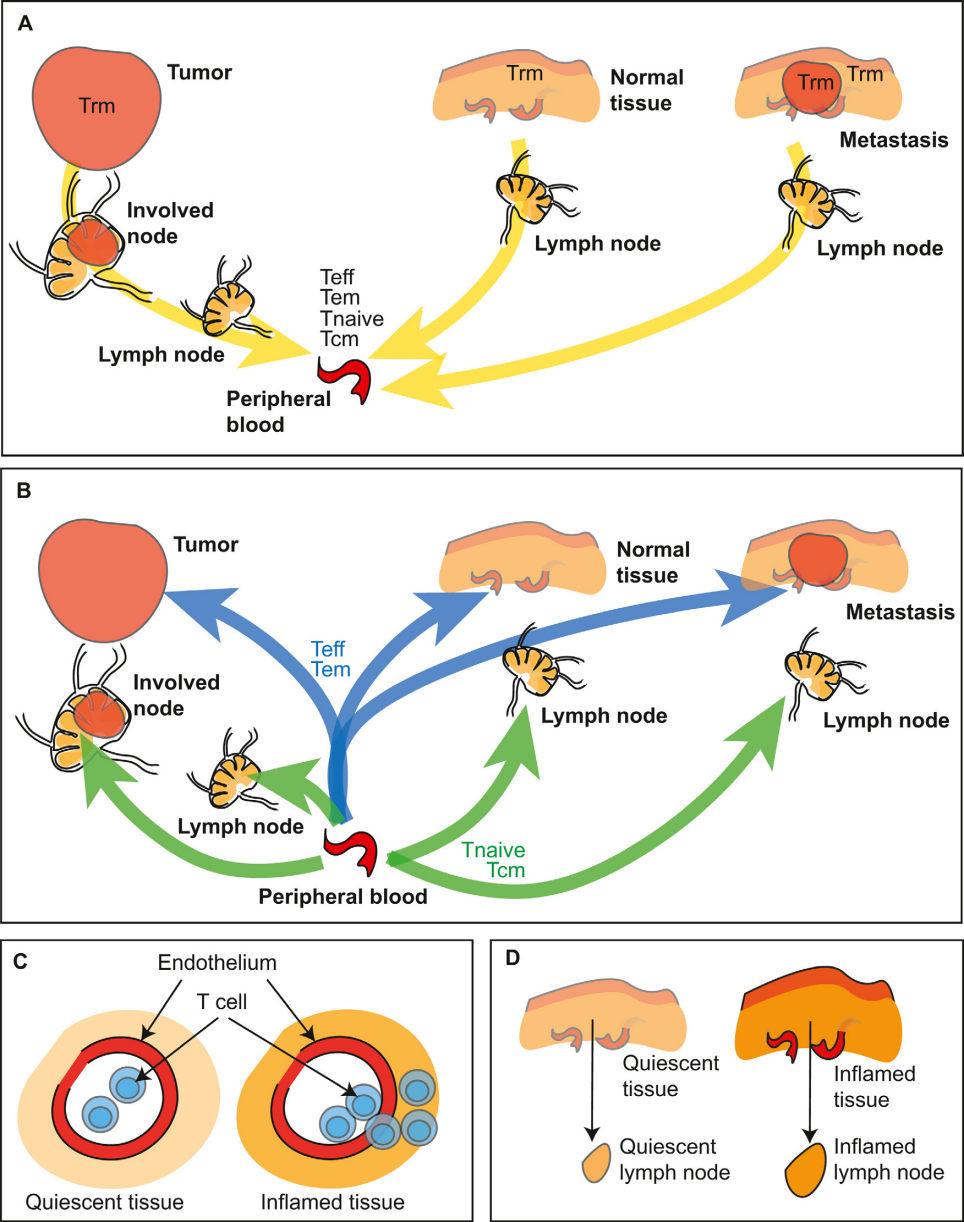
Trafficking of T cells is not antigen-directed. A) T cells that exit the tumor pass through the lymphatic chain before re-entering the peripheral blood at the thoracic duct. Similar recirculation pathways return all T cell populations except tissue resident memory cells (Trm) to the peripheral circulation. B) Once in the peripheral blood, effector cells and effector memory cells (Teff, Tem) can be recruited back to the tumor, they may enter normal tissues or metastatic sites according to local inflammatory conditions. Naive T cells and central memory T cells (Tnaive, Tcm) can directly enter lymph nodes and recirculate without entering peripheral tissues. C) T cell entry to any particular tumor, tissue or lymph node is more likely if there is local inflammation that results in upregulation of adhesion molecules on the vasculature and chemokines. D) Inflammation in a tissue site can be propagated via the draining lymphatics to increase entry of T cells to the draining lymph node. Together, these features ensure additional surveillance of inflamed tissues and lymph nodes by recirculating T cells, but relative ignorance of tumors and metastases that are not inflamed.

As with primary tumors, tissue resident memory cells can be detected in metastatic tumors, [63] suggesting that each tumor generates its own population of tumor-specific cells. In melanoma patients distinct metastases within an individual patient had a high degree of overlap in neoantigen targets and TCR sequences, [178] where 11 TCR sequences accounted for 90-99% of the tumor specificity in one patient. [178] These data suggest similar T cell clones may be found in metastases and may be controlling the distant tumors independently of the primary. This is supported by experimental evidence showing that there can be wide variations in the degree of immune infiltrate of different metastases within a single individual. [179] Using an immunoscore approach to assess the infiltrate of primary and metastatic tumors, investigators demonstrated that there was no link between the immunoscore of the primary tumor and that of metastases in the same patient, and also wide variation between the immunoscore of the different metastases within a patient. [179] However, there was evidence of immunoediting in the metastases that had higher immunoscores. [179] These data indicate that metastases may enrich for tumor specific T cells and both the primary site and the metastatic sites may benefit from recirculation of specific T cells between locations to keep all tumors under control (Fig. 5a-b). Moreover, given that recruitment into peripheral sites and lymphatic systems is regulated by inflammation (Fig. 5c-d), therapies that alter inflammation at metastatic sites have the potential to dramatically impact the visibility of distant tumors to T cells generated following primary tumor treatment.

### Summary of the impact of radiation on T cells in their various locations

Together, these data indicate that radiation can kill T cells in the treatment field, whether in the tumor, lymph nodes, or peripheral blood, and suggest that loss of these cells can impact patient outcome. These T cells are important for control of residual disease inside the field and control of unirradiated distant disease, [180,181] so patients that avoid systemic T cell loss see an improved benefit of treatment. [9] Since T cell loss in the field is unavoidable, these data imply either that radiation-mediated loss of T cells in the field is not sufficient to eliminate all activity, that cells outside of the field are critical to responses, or a combination of both. Certainly, the former is true. Not all T cells in the field are killed by radiation therapy, even though the doses to which the cells are exposed are nominally sufficient to kill them *ex vivo*. While control of residual disease in the field is an important role for T cells following radiation therapy, it is important to understand the relationships between the immune environment of the treated tumor, that of distant tumors, and how immune responses may be propagated between sites following treatment.

### Revisiting the Paradox

In view of these discussions, we should revisit the apparent paradox to explore whether each statement remains impactful given the current understanding in the field.

#### T cells are needed for radiation's efficacy

It certainly seems true that T cells are needed to amplify the efficacy of radiation therapy, and particularly to generate tumor responses outside the field. However, current treatment plans may not be optimally designed to exploit T cells, and so radiation may have been optimized to be an effective killer of cancer cells without help from T cells. A clear example is that extending the field to kill cancer cells in lymph nodes takes priority over any potential loss of immune responses following radiation. At present, this is likely appropriate. Nevertheless, in the short term there are clinical scenarios where sparing lymph nodes is being explored, for example in neoadjuvant treatment of HPV^+^ HNSCC, [182] and in the long term we may be able to better protect tumor-specific T cells from the negative effects of radiation through advanced treatments, timings, or combinations.

#### Tumor-specific T cells are enriched in the field of treatment

This is likely true, whether these cells are in the tumor or in lymph nodes within the field. Yet these are not the only tumor-specific T cells in the patient. There are low frequencies of these cells circulating through the peripheral blood, recirculating through distant tissues, and resident in distant metastases. The interchange between these populations, and how this is impacted by radiation is poorly understood. Non-exhausted stem-like T cells that are outside the suppressive environment of the tumor have the potential to be reactivated on therapy, in particular when combined with therapies that lower the threshold for their reactivation. [162,183] These T cells may be dependent on effective antigen cross-presentation in lymph nodes since they may be more common in the lymph nodes than in the tumor. Importantly, these T cells represent an opportunity to reinvigorate systemic immunity following radiation therapy.

#### Radiation kills T cells in the treatment field

This is also likely true, but surprisingly when considering the effect of radiation on T cells within the tumor this may be of least importance. T cells in irradiated tumors are surprisingly resistant to radiation as compared to circulating T cells, [13] even when recirculation is blocked multiple T cell populations can proliferate and repopulate a tumor treated with high radiation doses, [74] and radiation therapy and immunotherapy can cure tumors without needing repopulation by recirculating cells. [66,67] However, these preclinical studies were performed with single doses of radiation or short courses. We do not fully understand the consequence of fractionated radiation therapy on in-field T cells. While patients with solid tumors commonly receive 35 fractions, we know that in patients with lymphoma fractionated radiation therapy can eliminate lymphocytes in 15-20 fractions. [184] While lymphoma cells are non-identical to normal lymphocytes, it suggests that continued radiation delivery has the potential to eliminate lymphocytes that may be resident in the field long before completion of treatment. In preclinical models of cancer, standard fractionation over 6 weeks is rarely tested in combination with immunotherapy. In part there are practical limitations on such approaches, since the fast tumor growth rate and relative radioresistance of mice and murine tumors means that few tumors are effectively controlled with 1.8Gy/day, and most control-treated tumors will have grown beyond allowable limits before radiation treated groups have completed treatment. However, as discussed above, extending radiation fractionating into the effector phase of the immune response can has been shown to have no impact, [154] to impair the anti-tumor immune response, [151] or to improve the anti-tumor immune response. [152] There are a range of ongoing discussions on the optimum dose and fractionation scheme for synergy with immune mechanisms, with no clear consensus at present. However, hypofractionation for lymphocyte preservation [10] may be as important a consideration as optimum effects on the local immune environment, if lymphocytes are the critical final effector mechanism.

It may be possible to minimize potential immunological injury when delivering radiation therapy. For example, when immunotherapies are combined with radiation therapy, we may not need to deliver as high a radiation dose for similar efficacy. By calculating the biological equivalent dose (BED), we can estimate and compare the effective dose received using different dose and fractionation schemes. [153] Milas et al. used TCD_50_ analysis (dose to produce 50% tumor cures) when combining intratumoral CpG administration with radiation or radiation alone in immunocompetent mice. [185] The authors demonstrated a shift in TCD_50_ from approximately 40Gy x1 (BED_10_ of 200Gy) with radiation alone to approximately 20Gy x1 (BED_10_ of 60Gy) when combined with CpG. [185] When radiation was separated into 10 fractions the TCD_50_ shifted from 8-9Gy x10 (BED_10_ 144-154Gy) for radiation alone to 2-3Gy x10 (BED_10_ 24-39Gy) when combined with CpG. [186] Thus, the combination with immunotherapy may allow us to drop the radiation dose and proportionately spare T cells. However, these lower doses are still in the range likely to cause direct cytotoxicity to many T cells. [68] It seems likely that issues beyond dose are important to avoid damage of critical T cells.

Together, when we consider the paradox of radiation and T cells, it seems that T cells in the tumor are remarkably effective despite being irradiated. It is also surprising that T cells that are outside the tumor (but perhaps in the field) are the cells most impacted by current treatment plans. By considering the location of critical T cells and their contribution to local versus systemic responses, we may be able to design treatments that optimize the immune consequence of radiation therapy.

## CRediT authorship contribution statement

**Michael J. Gough:** Conceptualization, Writing – original draft, Visualization. **Marka R. Crittenden:** Conceptualization, Writing – review & editing.
